# Supplementary material for: Adherence and Persistence to Biological Drugs for Psoriasis: Systematic Review with Meta-Analysis
Source: J Clin Med. 2022 Mar 9;11(6):1506. doi: 10.3390/jcm11061506 (PMC8953825; doi:10.3390/jcm11061506)

Summary

Supplementary Material S1. Search strategy

Table S1. Summary of findings of included studies

Table S2. Risk of bias for prevalence studies

Figure S1. Forest plot of mean adherence

Table S3. Qualitative description of the main reasons for biological drug discontinuation or switching.

Figure S2. Forest plot of proportions of adherent patients with psoriasis to biological drugs stratified by study design

Figure S3. Forest plot of proportions of adherent patients with psoriasis to biological drugs stratified by type of patients

Figure S4. Forest plot of proportions of adherent patients with psoriasis to biological drugs stratified by risk of bias

Figure S5. Forest plot of proportions of persistent patients with psoriasis to biological drugs stratified by type of patients

Figure S7. Forest plot of proportions of persistent patients with psoriasis to biological drugs stratified by risk of bias

Supplementary Material S1. Search strategy

PUBMED: ((psoriatic AND disease*) OR psoriasis) AND (adherence OR compliance OR persistence OR switching OR (drug AND survival)) AND (etanercept OR ustekinumab OR adalimumab OR infliximab OR ixekizumab OR secukinumab OR biologic*)

EMBASE: ((psoriatic AND ('diseases'/exp OR 'diseases')) OR 'psoriasis'/exp OR 'psoriasis') AND ('adherence'/exp OR 'adherence' OR 'compliance'/exp OR compliance OR 'persistence'/exp OR 'persistence' OR 'switching'/exp OR 'switching' OR (('drug'/exp OR 'drug') AND ('survival'/exp OR 'survival'))) AND ('etanercept'/exp OR 'etanercept' OR 'ustekinumab'/exp OR 'ustekinumab' OR 'adalimumab'/exp OR 'adalimumab' OR 'infliximab'/exp OR 'infliximab' OR 'ixekizumab'/exp OR 'ixekizumab' OR 'secukinumab'/exp OR 'secukinumab' OR biologic*)

Table S1. Summary of findings of included studies

Study name	Outcome	Study design	Age in years, mean ± SD (range)	Women, %	Study population	Adherence or persistence definition/calculation	Included in the overall adherence/persistence	Included in the subgroup analysis (biological drugs)	Included in the subgroup analysis (biologic-naïve or experienced patients)	Risk of bias
Armstrong, 2018	Adherence (survey)	Retrospective cohort study	55.8 ± 15.7	52.4%	Patients with moderate-to-severe psoriasis	"Patients reported to be adherent to treatment by directly answering to a specific question"	Yes	No	No	7
Armstrong, 2018	Persistence	Retrospective cohort study	55.8 ± 15.7	52.4%	Patients with moderate-to-severe psoriasis	"Number of patients who did not switch therapy during the study period"	Yes	No	No	7
Bergman, 2020	Good adherence (PDC)	Retrospective cohort study	46.0 ± 13.1	60.4%	Patients affected by psoriasis, RA, PsA, Crohn's disease who were naive to ADA	"PDC ≥ 80%, defined as the number of days covered by the index medication during the 12-month follow-up period divided by 365 days"	No (Number of adherent psoriasis patients not available)	No	No	10
Bhosle, 2006	Adherence	Retrospective cohort study	41.0 ± 11.4	58.6%	Patients with psoriasis vulgaris	"MPR ≥ 80%, calculated as the days of supply for dispensed prescriptions for biologics divided by the number of days between these prescription refills"	Yes	No	No	9
Blauvelt, 2020a	Good adherence (MPR)	Retrospective cohort study	47.6 ± 12.8	44.9%	Patients with psoriasis	"MPR ≥ 80%, calculated as the total days' supply during the follow-up period divided by the length of the follow-up period"	Yes	Yes	No	8
Blauvelt, 2020a	Persistence	Retrospective cohort study	47.6 ± 12.8	44.9%	Patients with psoriasis	"No treatment gap ≥60 days"	Yes	Yes	No	8
Blauvelt, 2020b	Good adherence (MPR)	Retrospective cohort study	50.0 ± 12.0	48.3%	Patients with psoriasis	"MPR ≥ 80%, calculated as the total days' supply during the follow-up period divided by the length of the follow-up period"	Yes	Yes	No	8

Blauvelt, 2020b	Persistence	Retrospective cohort study	50.0 ± 12.0	48.3%	Patients with psoriasis	"No treatment gap ≥60 days"	Yes	Yes	No	8
Bonafede, 2012	Persistence	Retrospective cohort study	44 ± 11	68.1%	Anti TNF-α users affected by psoriasis, PsA, RA, PsA	"No treatment gap ≥45 days after the end of estimated clinical benefit for that treatment, which was assumed to be 7 days for ETN, 14 days for ADA, and 56 days for INF"	Yes	Yes	Yes	9
Bonafede, 2013	Persistence	Retrospective cohort study	45.6 ± 12.5	43.8%	Patients with psoriasis, PsA, or both	"Number of days from the index date to either the date of a switch to another biologic drug or a gap in therapy ≥ 45 days"	Yes	Yes	Yes	9
Cao, 2014	Persistence	Retrospective cohort study	49.0 ± 12.5	46.1%	Patients with moderate-to-severe psoriasis	"Absence of a medication gap of 130 days or more following the end of the days' supply of the previous prescription or administration"	Yes	Yes	No	8
Chan, 2013	Adherence (scale)	Cross-sectional	(16-99)	50.0%	Patients with psoriasis	"Therapy use, assessed on a Likert scale ranging from always (100%) to never (0%)"	Yes	No	No	6
Chastek, 2013	Persistence	Retrospective cohort study	43.0 ± 12.0	45.9%	Biologic-naïve patients with psoriasis	"Continuous use of index biologic drug without a gap in therapy ≥60 days. Gaps in index therapy were identified as the time between the run-out of a fill until the date of the next fill"	Yes	Yes	Yes	9
Chastek, 2016	Persistence	Retrospective cohort study	(18-63)	59.2%	Patients with diagnosis of moderate-to-severe psoriasis, RA, PsA and/or AS	"Absence of switching from their index biologic or have a gap in therapy of at least 45 days at any time during the 1-year follow-up"	Yes	Yes	Yes	9

Clemmensen, 2011	Persistence	Retrospective cohort study	43.1 ± 13.0	49.3%	Patients with a diagnosis of psoriasis vulgaris	"Number of patients (%) who did not discontinue therapy"	Yes	Yes	No	9
Conesa-Nicolàs, 2020	Persistence	Retrospective cohort study	49.8 ± 12.6	47.1%	Patients affected by psoriasis, PsA, AS	"Patients who continued their treatment during follow-up"	Yes	Yes	No	7
Davila-Seijo, 2016	Persistence	Prospective cohort study	48.3 ± 13.5	39.5%	Patients with moderate-to-severe psoriasis	"Patients who did not discontinue therapy"	No (Number of adherent psoriasis patients not available)	No	No	7
Degli Esposti, 2014	Good adherence (PDC)	Retrospective cohort study	49.6 ± 14.6	53.0%	Patients affected by psoriasis, RA, Crohn's disease, ulcerative colitis, PsA or AS	"PDC ≥ 80%, calculated as sum of prescriptions coverage (days)/duration of the follow-up period (365 days) *100"	Yes	No	No	10
Degli Esposti, 2014	Persistence	Retrospective cohort study	49.6 ± 14.6	53.0%	Patients affected by psoriasis, RA, Crohn's disease, ulcerative colitis, PsA or AS	"Number of patients who did not interrupted treatment. Treatment interruption was defined as the absence of prescriptions in the last 3 months of observation period"	Yes	No	No	10
Dommasch, 2018	Good adherence (PDC)	Retrospective cohort study	48.2	44.5%	Patients affected by psoriasis	" PDC ≥ 80%. PDC is equals the number of days covered by prescription for the study drug divided by the number of days during which the patient was eligible"	Yes	Yes	Yes	10
Doshi, 2016	Good adherence (PDC)	Retrospective cohort study	60.7 ± 14.5	56.1%	Patients affected by plaque psoriasis	"PDC ≥ 80%, measured as the number of days covered with the index biologic divided by a fixed time interval (i.e., 365 days) from the date of index biologic therapy initiation"	Yes	Yes	Yes	10

Doshi, 2016	Persistence	Retrospective cohort study	60.7 ± 14.5	56.1%	Patients affected by plaque psoriasis	"No treatment gap ≥90 days during the 12-month follow-up period"	Yes	Yes	Yes	10
Egeberg, 2019	Persistence	Retrospective cohort study	47.5 ± 14.0	39.1%	Patients with moderate-to-severe plaque psoriasis	"Patients who did not discontinue biological therapy"	Yes	Yes	Yes	7
Esposito, 2013	Persistence	Retrospective cohort study	49 ± 13.1	34.5%	Adult patients with plaque psoriasis or PsA	"Patients who did not discontinue (absence of treatment interruptions > 90 days)"	Yes	Yes	Yes	8
Esposito, 2014	Persistence	Retrospective cohort study	(18-99)	/	Adult patients with plaque psoriasis	"Patients (%) still on treatment at the end of the observation period"	Yes	Yes	Yes	7
Feldman, 2015	Persistence	Retrospective cohort study	45.7 ± 12.9	44.5%	Patients with diagnosis of moderate-to-severe psoriasis	"No treatment gaps (time between the preceding fill and the time to the next refill) that exceeded the specified time thresholds (4 weeks for ETN, 8 weeks for ADA, and 18 weeks for UST)"	Yes	Yes	Yes	9
Feldman, 2019	Good adherence (PDC)	Retrospective cohort study	42.6 ± 12.0	52.3%	Psoriasis patients with or without concomitant metabolic conditions	"PDC ≥ 80%, calculated using the number of days the prescription refill claims should "cover" divided by the number of days over the observation period"	Yes	Yes	Yes	10
Ferrieres, 2019	Persistence	Retrospective cohort study	48.8 (28-78)	34.1%	Patients with moderate-to-severe psoriasis (plaque psoriasis or palmoplantar pustular psoriasis or PsA)	"Patients continuing treatment during follow-up"	Yes	Yes	No	5
Gendelman, 2018	Good adherence (MPR)	Retrospective cohort study	33.7 ± 12.8	38.2%	Patients with psoriasis or RA or PsA or AS or Crohn's disease	"MPR ≥ 80%, measured as the ratio of the number of treatment days dispensed and the total number of days from first dispense to the	Yes	Yes	No	9

						last supply day of the last dispense in the follow-up period or until discontinuation ≥ 180 days (gap between dispenses)"			
Gniadecki, 2011	Persistence	Retrospective cohort study	45 (9-77)	32.9%	Patients with psoriasis vulgaris	"Patients who are still on drug after 4 years of treatment"	Yes	Yes	No	9
Gniadecki, 2015	Persistence	Retrospective cohort study	45.2 ± 14.2	35.5%	Patients with psoriasis vulgaris	_	No (Number of adherent psoriasis patients not available)	No	No	7
Hendrix, 2020	Persistence	Retrospective cohort study	43.7 ± 11.6	52.5%	Patients with plaque psoriasis	"Absence of discontinuation, that is not refilling a drug or switching to a different targeted immunomodulator during the permissible gap of 90 days"	Yes	No	Yes	9
Higa, 2019	Persistence	Retrospective cohort study	46.1 ± 13.0	51.0%	Adult psoriasis patients	"Continuous use from initiation to a > 30-day gap between claims"	Yes	No	Yes	8
Howe, 2014	Persistence	Retrospective cohort study	44.0 ± 11.4	64.0%	Patients with diagnosis of psoriasis, RA, PsA, AS, or combination of these diseases	"Patients who continued on their index biologics without a ≥45-day gap in therapy until the end of the follow-up period and without a switch to another biologic agent"	Yes	Yes	Yes	10
Hsu, 2016	Good adherence (MPR)	Retrospective cohort study	43.5 ± 14.5	47.4%	Patients with psoriasis vulgaris	"MPR ≥ 80%, calculated by dividing the actual use of medication by the stipulated use"	Yes	Yes	No	9
Ichiyama, 2018	Adherence (scale)	Cross-sectional	57.9 ± 14.2	22.1%	Patients with psoriasis	"Adherence has been assessed using the eight-item Morisky Medication Adherence Scale (MMAS-8). High adherence: score = 8"	No (Number of adherent psoriasis patients not available)	No	No	7

Iskandar, 2018	Persistence	Retrospective cohort study	46.3 ± 12.8	41.6%	Patients with psoriasis who had failed a first biologic	"Patients who were still on treatment on their first-line biologic treatment (gap < 90 days)"	Yes	No	Yes	7
Jacobi, 2016	Persistence	Retrospective cohort study	47 (39-52)	35.8%	Patients with plaque psoriasis	"Patients who did not discontinue biologic therapy"	Yes	No	No	5
Khalid, 2014	Persistence	Retrospective cohort study	48.7 ± 12.9	40.8%	Adult patients diagnosed with psoriasis	"Patients who received biologic therapy continuously for the 12 months of observation (no gaps ≥ 68 days between administrations)"	Yes	No	Yes	9
Lee, 2018	Persistence	Retrospective cohort study	47.9 ± 11.9	50.4%	Adult patients with diagnosis of psoriasis and/or PsA	"Persistence was determined based on a > 90-day gap. Days' supplies were defined according to each biologic's dosing schedule: ETN: 7 days; ADA: 14 days; UST: 90 days; INF: 60 days"	Yes	No	No	10
Li, 2014	Adherence (PDC)	Retrospective cohort study	44.3 ± 12.9	44.4%	Patients with psoriasis	"Adherence has been calculated using PDC. Adherent patients: PDC ≥ 75%"	Yes	No	Yes	10
Magis, 2017	Persistence	Retrospective cohort study	51.7 ± 12.6	35.6%	Patients with moderate-to-severe plaque psoriasis	"Patients who continued treatment for more than 12 months"	Yes	Yes	No	7
Mahlich, 2019	Persistence	Retrospective cohort study	46.6 ± 13.2	31.8%	Biologic-naive psoriasis patients	"Patients who did not discontinue therapy. Discontinuation has been defined as gap>60 days after the end of supply of the index biologic agent or switching to another treatment during follow-up"	Yes	Yes	Yes	10

Malatestinic, 2017	Good adherence (MPR)	Retrospective cohort study	56.3 ± 16.1	59.4%	Patients with moderate-to-severe psoriasis	"MPR≥ 80%, calculated by dividing total days' supply of filled biologic over the number of days following initiation"	Yes	Yes	No	10
Malatestinic, 2017	Persistence	Retrospective cohort study	56.3 ± 16.1	59.4%	Patients with moderate-to-severe psoriasis	"No treatment gap ≥90 days during the 12-month follow-up period"	Yes	No	No	10
Marshall, 2018	Good adherence (MPR)	Retrospective cohort study	(18-99)	55.0%	Adult patients affected by psoriasis, RA, SA, Crohn's disease or ulcerative colitis	"MPR > 80% (sum of unique days of medication supplied over the 36-month study period, divided by the total number of days between the first and last transactions)"	No (Number of adherent psoriasis patients not available)	No	No	9
Menter, 2016	Persistence	Prospective cohort study	46.9 ± 14.3	43.3%	Patients affected by psoriasis	"Patients who did not stopped/switched biologic therapy on registry"	Yes	Yes	Yes	9
Mercadal-Orfilia, 2016	Persistence	Retrospective cohort study	48.4 ± 14.2	43.0%	Patients affected by moderate-to-severe psoriasis	"Persistence rates were reckoned tacking into account the current total days of therapy comparing posology with supplied dose"	Yes	Yes	Yes	9
Mocanu, 2019	Persistence	Retrospective cohort study	58.9 (9-82)	42.9%	Patients affected by moderate-to-severe psoriasis	"Patients who did not discontinue therapy"	Yes	Yes	No	8
Murage, 2019a	Good adherence (MPR)	Retrospective cohort study	47.6 ± 11.7	45.4%	Adult patients affected by moderate-to-severe psoriasis	"MPR ≥ 80% (number of days of therapy during the follow-up period divided by 360 days)"	Yes	Yes	Yes	10
Murage, 2019a	Persistence	Retrospective cohort study	47.6 ± 11.7	45.4%	Adult patients affected by moderate-to-severe psoriasis	"Persistence was defined as time to discontinuation of biologics with a 90-day permissible gap"	Yes	Yes	Yes	10
Murage, 2019b	Persistence	Retrospective cohort study	49.0 ± 10.9	44.2%	Adults diagnosed with psoriasis having ≥1 IXE claim	"Patients not achieving the discontinuation threshold (using a 60-day or 90-day	Yes	Yes	No	9

						gap) from the index date"				
Onsun, 2020	Persistence	Retrospective cohort study	48.2 ± 14.6	46.2%	Patients affected by moderate-to-severe plaque psoriasis	"Number of patients who continued treatment during follow-up"	Yes	Yes	No	9
Pogacsas, 2017	Persistence	Retrospective cohort study	49.3 ± 14.3	38.7%	Psoriasis patients	"Number (%) of patients who were still on treatment after 1-3 years of follow-up"	Yes	No	No	9
Ross, 2016	Persistence	Prospective cohort study	49.5	39.7%	Patients with chronic plaque psoriasis	"Cumulative % of patients remaining on a treatment"	Yes	No	No	9
Ruiz-Villaverde, 2020	Persistence	Retrospective cohort study	47 ± 14.5	45.0%	Patients with moderate-to-severe plaque psoriasis	"Absence of discontinuation, categorized as primary or secondary failure with adverse events, disease improvement, or other reasons"	Yes	Yes	No	10
Sbidian, 2019	Persistence	Retrospective cohort study	48.6 ± 13.5	42.7%	Biologic naive adult patients with psoriasis	"Number of patients who did not discontinue therapy. Discontinuation has been defined as a period > 90 days without fulfilment of a prescription for the same treatment after the period covered by the previous prescription"	Yes	Yes	Yes	8
Sruamsiri, 2018	Persistence	Retrospective cohort study	47.1	18.1%	Patients with psoriasis	"Number of patients who did not discontinue therapy. Discontinuation has been defined as switching or gap > 90 consecutive days without the index biological therapy (non-biological therapy refill period) for ADA, INF, SECU or 150 days for UST"	Yes	Yes	Yes	10

Svedbom, 2014	Persistence	Retrospective cohort study	47.8 ± 13.0	48.2%	Adult patients with psoriasis	"Persistence was measured from the date the patient filled the index prescription until the end of the duration of the last prescription. Patients were defined as non-persistent if they had a gap > 60 days"	Yes	No	No	10
Thorneloe, 2017	Adherence (scale)	Cross-sectional	47.9 ± 12.8	42.9%	Adult patients with diagnosis of psoriasis	"Adherence has been calculated by using the Medication Adherence Report Scale (MARS). Patients were classified into adherent and non-adherent using a score of ≤ 38/40"	Yes	Yes	No	8
Umezawa, 2013	Persistence	Retrospective cohort study	53.6 ± 11.3	27.6%	Patients diagnosed with plaque psoriasis, PsA, generalized pustular psoriasis or psoriatic erythroderma	"Patients who continued treatment for up to 1 year"	Yes	Yes	No	9
Van den Reek, 2014	Persistence	Prospective cohort study	47.1 ± 12.5	44.8%	Patients with moderate-to-severe psoriasis	"Patients who continued treatment for up to 1 year and 4.5 year"	Yes	Yes	No	9
Verma, 2017	Persistence	Retrospective cohort study	53.0 ± 14.2	45.4%	Patients with plaque psoriasis	"Patients still on treatment after 1-5 years of treatment"	Yes	Yes	No	10
Wang, 2020	Adherence (survey)	Cross-sectional	39.1 ± 12.2	36.9%	Chinese patients with psoriasis	Adherence has been measured by a single question "During the epidemic, did you adhere to the medication or treatment prescribed by the physician?"	No (Number of adherent psoriasis patients not available)	No	No	8
Wu, 2020	Good adherence (MPR)	Retrospective cohort study	48.7-52.2	45.1%	Adult patients diagnosed with moderate-to-severe psoriasis	"MPR>80%, calculated as sum of days' supply for the index medication during the follow-up period divided by the number of days between the index date + 1 and the	Yes	Yes	Yes	10

						last observed fill date + days' supply of the last fill"				
Wu, 2020	Persistence	Retrospective cohort study	48.7-52.2	45.1%	Adult patients diagnosed with moderate-to-severe psoriasis	"Persistence has been defined as ≥292 days of continuous use. The maximum allowed gap was 14 days for ADA, 7 days for ETN, 28 days for SECU, 84 days for UST"	Yes	Yes	Yes	10
Yeung, 2013	Persistence	Cross-sectional	49 (37-60)	48.6%	Patients with moderate-to-severe plaque psoriasis	_	No (Number of adherent psoriasis patients not available)	No	No	8
Yiu, 2020	Persistence	Prospective cohort study	45 (35-55)	40.7%	Patients with chronic plaque psoriasis	Authors reported the number of patients who discontinued treatment. They defined discontinuation as any gap in treatment > 90 days	Yes	Yes	No	10
Zagni, 2020	Persistence	Retrospective cohort study	50.2 ± 14.3	39.6%	Adult patients with psoriasis, PsA or AS diagnosis and ≥1 prescription of biologic drugs	"Presence of the biologic index therapy during the last trimester of follow-up".	Yes	Yes	No	8
Zschocke, 2017	Adherence (scale)	Cross-sectional	47.8 ± 12.5	34.2%	Patients with moderate-to-severe active plaque psoriasis	"A sum score was calculated for five items contained in the Physician Questionnaire Part Ib. A sum score of ≤ 3 was defined as indicating adherence"	Yes	No	No	8

* Abbreviations: ETN: etanercept; ADA: adalimumab; INF: infliximab; IXE: ixexizumab; UST: ustekinumab; SECU: secukinumab; EFA: efalizumab; ALE: alefacept; PsA: psoriatic arthritis; AS: ankylosing spondylitis; RA: rheumatoid arthritis; PDC: proportion of days covered; MPR: medication possession ratio

Table S2. Risk of bias for prevalence studies

Risk of Bias – Prevalence Studies											
First Author	External validity				Internal Validity						
	1. A close representation	2. True or close representation	3. Random selection	4. Non-response bias minimal	5. Directly from the subjects	6. Acceptable case definition	7. Measured was reliability and validity	8. Same mode of data collection	9. Length of the shortest prevalence period	10. Numerator(s) and denominators) appropriate	11. Summary
Armstrong et al. 2018	YES	YES	NO	YES	YES	NO	NO	YES	YES	YES	7
Bergman et al. 2020	YES	YES	YES	YES	YES	YES	YES	YES	YES	YES	10
Bhosle et al. 2006	NO	YES	YES	YES	YES	YES	YES	YES	YES	YES	9
Blauvelt et al. 2020a	NO	YES	YES	YES	YES	YES	YES	YES	NO	YES	8
Blauvelt et al. 2020b	NO	YES	YES	YES	YES	YES	YES	YES	NO	YES	8
Bonafede et al. 2012	NO	YES	YES	YES	YES	YES	YES	YES	YES	YES	9
Bonafede et al. 2013	NO	YES	YES	YES	YES	YES	YES	YES	YES	YES	9
Cao et al. 2014	NO	YES	YES	YES	YES	YES	YES	YES	NO	YES	8
Chan et al. 2013	YES	YES	YES	NO	YES	YES	NO	YES	NO		6
Chastek et al. 2012	NO	YES	YES	YES	YES	YES	YES	YES	YES	YES	9
Chastek et al. 2016	NO	YES	YES	YES	YES	YES	YES	YES	YES	YES	9
Clemmensen et al. 2010	YES	YES	YES	YES	YES	NO	YES	YES	YES	YES	9
Conesa et al. 2020	NO	YES	YES	YES	NO	YES	NO	YES	YES	YES	7
Davila et al. 2016	YES	NO	YES	YES	YES	NO	NO	YES	YES	YES	7
Degli Esposti et al. 2014	YES	YES	YES	YES	YES	YES	YES	YES	YES	YES	10
Dommasch et al. 2018	YES	YES	YES	YES	YES	YES	YES	YES	YES	YES	10
Doshi et al. 2016	YES	YES	YES	YES	YES	YES	YES	YES	YES	YES	10
Egeberg et al. 2019	YES	YES	YES	YES	NO	NO	YES	YES	NO	YES	7
Esposito et al. 2013	NO	YES	YES	YES	YES	YES	YES	YES	NO	YES	8
Esposito et al. 2014	NO	YES	YES	YES	YES	NO	YES	YES	NO	YES	7
Feldman et al. 2015	NO	YES	YES	YES	YES	YES	YES	YES	YES	YES	9

Feldman et al. 2019	YES	YES	YES	YES	YES	YES	YES	YES	YES	YES	10
Ferrieres et al. 2019	NO	YES	NO	YES	YES	NO	NO	YES	NO	YES	5
Gendelman et al. 2018	YES	YES	YES	YES	YES	YES	YES	YES	NO	YES	9
Gniadecki et al. 2011	YES	YES	YES	YES	YES	NO	YES	YES	YES	YES	9
Gniadecki et al. 2015	YES	NO	YES	YES	YES	NO	YES	YES	NO	YES	7
Hendrix et al. 2020	NO	YES	YES	YES	YES	YES	YES	YES	YES	YES	9
Higa et al. 2019	NO	YES	YES	YES	YES	NO	YES	YES	YES	YES	8
Howe et al. 2014	YES	YES	YES	YES	YES	YES	YES	YES	YES	YES	10
Hsu et al. 2016	YES	NO	YES	YES	YES	YES	YES	YES	YES	YES	9
Ichiyama et al. 2018	NO	NO	YES	YES	YES	YES	YES	YES	NO	YES	7
Iskandar et al. 2018	NO	NO	YES	YES	YES	NO	YES	YES	YES	YES	7
Jacobi et al. 2016	NO	NO	YES	YES	YES	NO	NO	YES	NO	YES	5
Khalid et al. 2014	YES	YES	YES	YES	YES	NO	YES	YES	YES	YES	9
Lee et al. 2018	YES	YES	YES	YES	YES	YES	YES	YES	YES	YES	10
Li et al. 2014	YES	YES	YES	YES	YES	YES	YES	YES	YES	YES	10
Magis et al. 2017	YES	NO	YES	YES	YES	NO	NO	YES	YES	YES	7
Mahlich et al. 2019	YES	YES	YES	YES	YES	YES	YES	YES	YES	YES	10
Malatestinic et al. 2017	YES	YES	YES	YES	YES	YES	YES	YES	YES	YES	10
Marshall et al. 2018	YES	YES	YES	YES	YES	YES	YES	YES	YES	NO	9
Menter et al. 2016	YES	YES	YES	YES	YES	NO	YES	YES	YES	YES	9
Mercadal et al. 2016	NO	YES	YES	YES	YES	YES	YES	YES	YES	YES	9
Mocanu et al. 2019	YES	YES	YES	YES	YES	YES	YES	YES	NO	NO	8
Murage et al. 2018	YES	YES	YES	YES	YES	YES	YES	YES	YES	YES	10
Murage et al. 2019	YES	YES	YES	YES	YES	YES	YES	YES	NO	YES	9
Onsun et al. 2020	YES	YES	YES	YES	YES	NO	YES	YES	YES	YES	9
Pogacsas et al. 2017	YES	YES	YES	YES	YES	NO	YES	YES	YES	YES	9
Ross et al. 2016	YES	YES	YES	YES	YES	YES	YES	YES	YES	NO	9
Ruiz-Villaverde et al. 2020	YES	YES	YES	YES	YES	YES	YES	YES	YES	YES	10
Sbidian et al. 2019	NO	YES	YES	YES	YES	YES	YES	YES	NO	YES	8

Sruamsiri et al. 2018	YES	YES	YES	YES	YES	YES	YES	YES	YES	YES	10
Svedbom et al. 2014	YES	YES	YES	YES	YES	YES	YES	YES	YES	YES	10
Thorneloe et al. 2014	YES	YES	YES	YES	YES	NO	YES	YES	NO	YES	8
Umezawa et al. 2013	YES	YES	YES	YES	YES	NO	YES	YES	YES	YES	9
Van der Reek et al. 2014	YES	YES	YES	YES	YES	NO	YES	YES	YES	YES	9
Verma et al. 2017	YES	YES	YES	YES	YES	YES	YES	YES	YES	YES	10
Wang et al. 2020	YES	YES	YES	YES	YES	NO	YES	YES	NO	YES	8
Wu et al. 2020	YES	YES	YES	YES	YES	YES	YES	YES	YES	YES	10
Yeung et al. 2013	YES	YES	YES	YES	YES	NO	NO	YES	YES	YES	8
You et al. 2020	YES	YES	YES	YES	YES	YES	YES	YES	YES	YES	10
Zagni et al. 2020	YES	YES	YES	YES	YES	NO	YES	YES	NO	YES	8
Zschocke et al. 2017	YES	YES	YES	YES	YES	NO	NO	YES	YES	YES	8

Figure S1. Forest plot of mean adherence

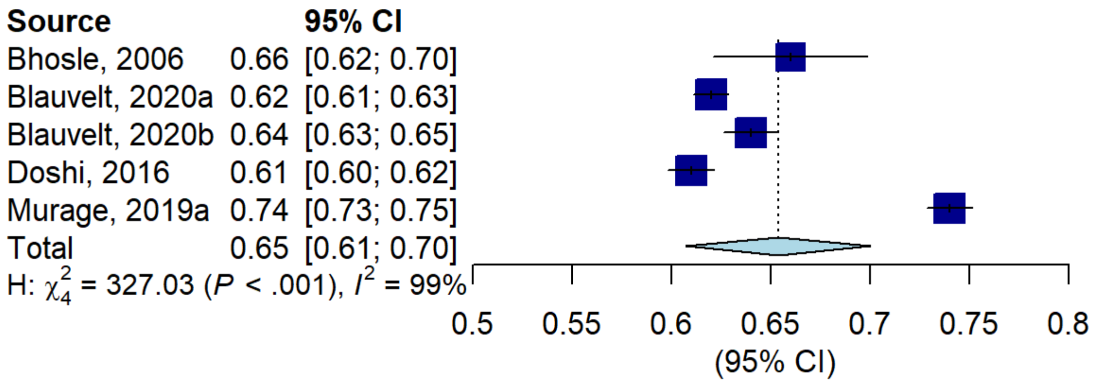

Table S3. Qualitative description of the main reasons for biological drug discontinuation or switching.

**Reasons**	**Number of studies**
Loss of efficacy and adverse events	8 for persistence (47.1%), 2 for adherence (100%)
Ineffectiveness	3 (17.6%) for persistence
Other reasons[1]	6 (35.3%) for persistence
Not reported	21 for persistence, 14 for adherence

[1]aggravation of psoriatic arthritis and swallowing disturbances; lack of efficacy, adverse events, and remission; adverse events and patient's choice; ineffectiveness and patient's choice; ineffectiveness and adverse events; loss of efficacy and remission.

Figure S2. Forest plot of proportions of adherent patients with psoriasis to biological drugs stratified by study design

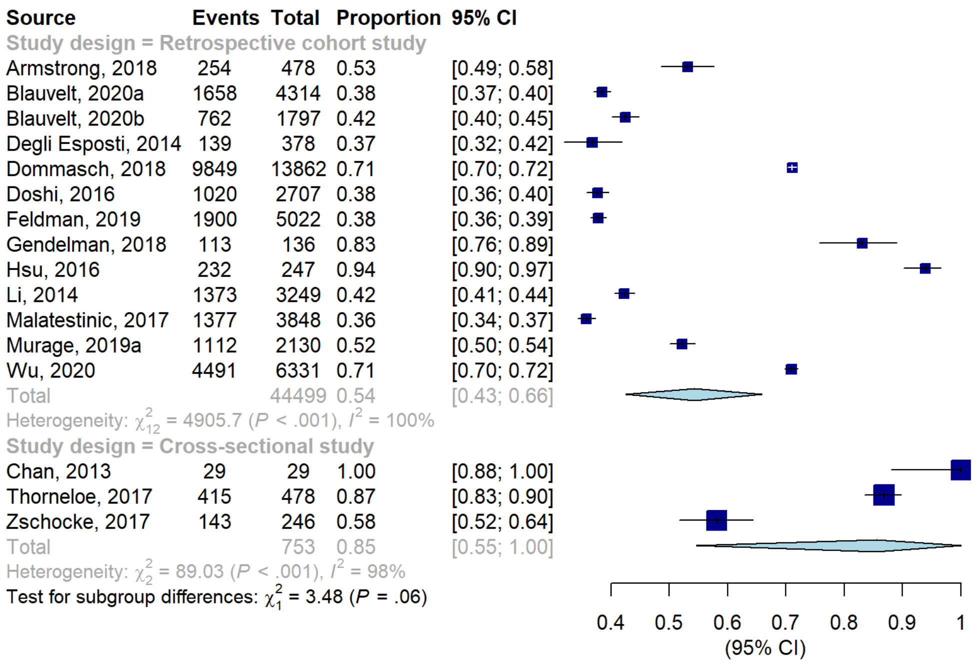

Figure S3. Forest plot of proportions of adherent patients with psoriasis to biological drugs stratified by type of patients

Source	Events	Total	Proportion	95% CI
naive_exp = Naive				
Dommasch, 2018	9849	13862	0.71	[0.70; 0.72]
Doshi, 2016	1020	2707	0.38	[0.36; 0.40]
Feldman, 2019	1900	5022	0.38	[0.36; 0.39]
Li, 2014	1373	3249	0.42	[0.41; 0.44]
Murage, 2019a	1112	2130	0.52	[0.50; 0.54]
Wu, 2020	4491	6331	0.71	[0.70; 0.72]
Total		33301	0.52	[0.39; 0.65]
Heterogeneity: $\chi^2_5 = 3107.32$ ($P < .001$), $I^2 = 100\%$				
naive_exp = NotSpecified				
Armstrong, 2018	254	478	0.53	[0.49; 0.58]
Blauvelt, 2020a	1658	4314	0.38	[0.37; 0.40]
Blauvelt, 2020b	762	1797	0.42	[0.40; 0.45]
Chan, 2013	29	29	1.00	[0.88; 1.00]
Degli Esposti, 2014	139	378	0.37	[0.32; 0.42]
Gendelman, 2018	113	136	0.83	[0.76; 0.89]
Hsu, 2016	232	247	0.94	[0.90; 0.97]
Malatestinic, 2017	1377	3848	0.36	[0.34; 0.37]
Thorneloe, 2017	415	478	0.87	[0.83; 0.90]
Zschocke, 2017	143	246	0.58	[0.52; 0.64]
Total		11951	0.66	[0.47; 0.82]
Heterogeneity: $\chi^2_9 = 1145.28$ ($P < .001$), $I^2 = 99\%$				
Test for subgroup differences: $\chi^2_1 = 1.39$ ($P = .24$)				

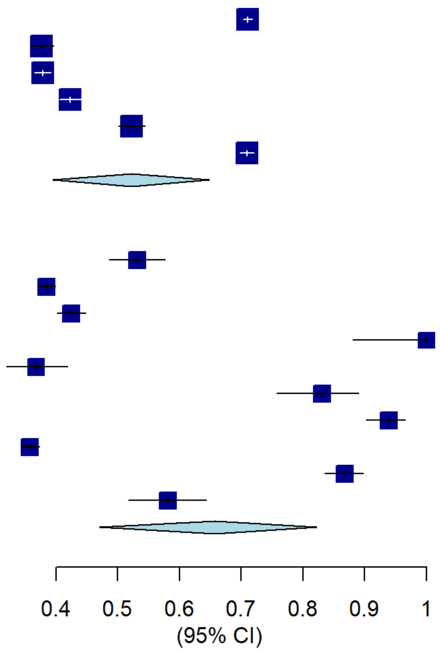

Figure S4. Forest plot of proportions of adherent patients with psoriasis to biological drugs stratified by risk of bias

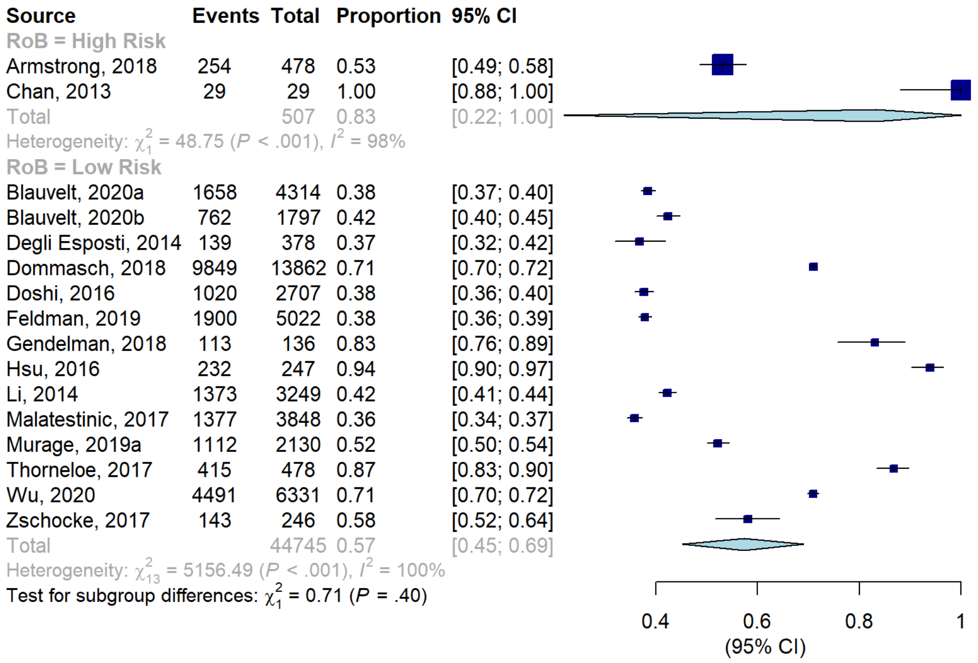

Figure S5. Forest plot of proportions of persistent patients with psoriasis to biological drugs stratified by type of patients

Source	Events	Total	Proportion	95% CI
naive_exp = Naive				
Bonafede, 2012	230	874	0.26	[0.23; 0.29]
Bonafede, 2013	1408	2775	0.51	[0.49; 0.53]
Chastek, 2013	341	827	0.41	[0.38; 0.45]
Chastek, 2016	657	2002	0.33	[0.31; 0.35]
Doshi, 2016	1489	2707	0.55	[0.53; 0.57]
Egeberg, 2019	123	158	0.78	[0.71; 0.84]
Esposito, 2013	472	650	0.73	[0.69; 0.76]
Esposito, 2014	128	152	0.84	[0.77; 0.90]
Feldman, 2015	1492	4309	0.35	[0.33; 0.36]
Hendrix, 2020	6310	17054	0.37	[0.36; 0.38]
Higa, 2019	1200	2161	0.56	[0.53; 0.58]
Howe, 2014	171	398	0.43	[0.38; 0.48]
Iskandar, 2018	3771	5999	0.63	[0.62; 0.64]
Khalid, 2014	108	169	0.64	[0.56; 0.71]
Mahlich, 2019	99	176	0.56	[0.49; 0.64]
Menter, 2016	790	1115	0.71	[0.68; 0.74]
Mercadal Orfilia, 2016	91	112	0.81	[0.73; 0.88]
Murage, 2019a	1611	2130	0.76	[0.74; 0.77]
Sbidian, 2019	10241	16545	0.62	[0.61; 0.63]
Sruamsiri, 2018	112	177	0.63	[0.56; 0.70]
Wu, 2020	2112	6331	0.33	[0.32; 0.35]
Total		66821	0.56	[0.49; 0.64]
Heterogeneity: χ^2_{20} = 5408.54 (P < .001), I^2 = 100%				
naive_exp = Experienced				
Chastek, 2016	1530	3080	0.50	[0.48; 0.51]
Hendrix, 2020	11332	37276	0.30	[0.30; 0.31]
Howe, 2014	203	383	0.53	[0.48; 0.58]
Menter, 2016	1573	2358	0.67	[0.65; 0.69]
Total		43097	0.50	[0.35; 0.65]
Heterogeneity: χ^2_{3} = 1638.78 (P < .001), I^2 = 100%				
naive_exp = NotSpecified				
Armstrong, 2018	4336	11767	0.37	[0.36; 0.38]
Blauvelt, 2020a	1900	4314	0.44	[0.43; 0.46]
Blauvelt, 2020b	872	1797	0.49	[0.46; 0.51]
Cao, 2014	814	1000	0.81	[0.79; 0.84]
Clemmensen, 2011	147	179	0.82	[0.76; 0.87]
Conesa-Nicolàs, 2020	27	54	0.50	[0.36; 0.64]
Degli Esposti, 2014	253	378	0.67	[0.62; 0.72]
Ferrieres, 2019	69	91	0.76	[0.66; 0.84]
Gniadecki, 2011	337	747	0.45	[0.42; 0.49]
Jacobi, 2016	9	67	0.13	[0.06; 0.24]
Lee, 2018	1429	2249	0.64	[0.62; 0.66]
Magis, 2017	120	174	0.69	[0.62; 0.76]
Malatestinic, 2017	8033	11067	0.73	[0.72; 0.73]
Mocanu, 2019	64	84	0.76	[0.66; 0.85]
Murage, 2019b	646	801	0.81	[0.78; 0.83]
Onsun, 2020	215	247	0.87	[0.82; 0.91]
Pogacsas, 2017	1007	1263	0.80	[0.77; 0.82]
Ross, 2016	33	58	0.57	[0.43; 0.70]
Ruiz-Villaverde, 2020	149	171	0.87	[0.81; 0.92]
Svedbom, 2014	287	506	0.57	[0.52; 0.61]
Umezawa, 2013	104	127	0.82	[0.74; 0.88]
van der Reek, 2014	88	116	0.76	[0.67; 0.83]
Verma, 2017	617	882	0.70	[0.67; 0.73]
Yiu, 2020	4930	6497	0.76	[0.75; 0.77]
Zagni, 2020	1453	1947	0.75	[0.73; 0.77]
Total		46583	0.67	[0.60; 0.74]
Heterogeneity: χ^2_{24} = 5961.35 (P < .001), I^2 = 100%				
Test for subgroup differences: χ^2_{2} = 6.01 (P = .05)				

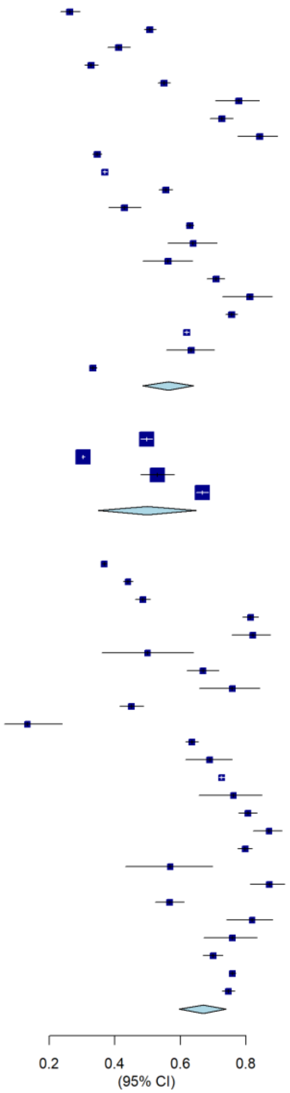

Figure S6. Forest plot of proportions of persistent patients with psoriasis to biological drugs stratified by study design

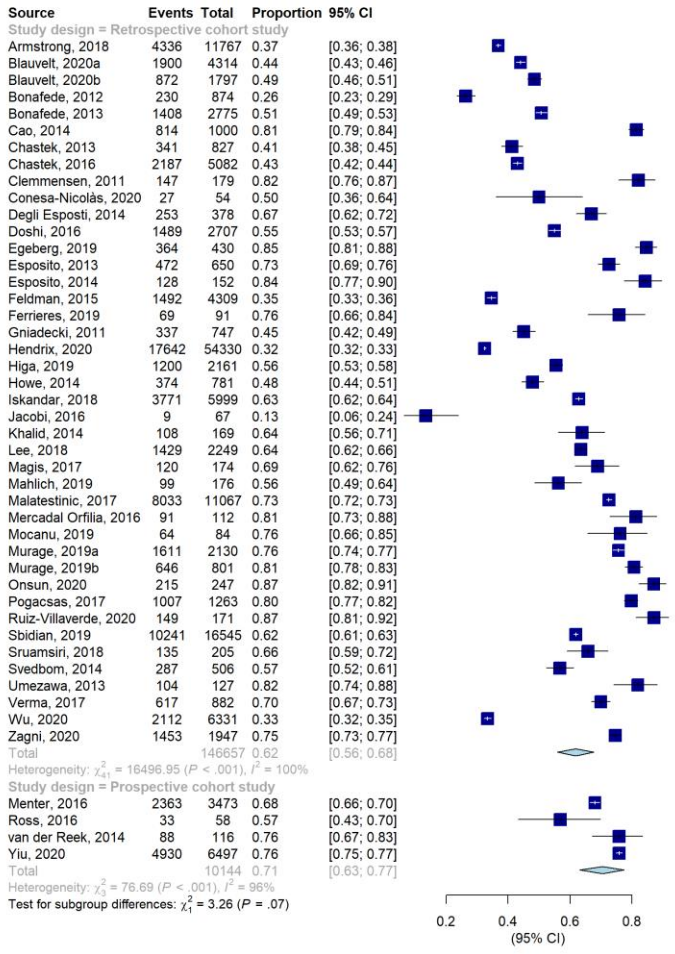

Figure S7. Forest plot of proportions of persistent patients with psoriasis to biological drugs stratified by risk of bias

Source	Events	Total	Proportion	95% CI
RoB = High Risk				
Armstrong, 2018	4336	11767	0.37	[0.36; 0.38]
Conesa-Nicolàs, 2020	27	54	0.50	[0.36; 0.64]
Egeberg, 2019	364	430	0.85	[0.81; 0.88]
Esposito, 2014	128	152	0.84	[0.77; 0.90]
Ferrieres, 2019	69	91	0.76	[0.66; 0.84]
Iskandar, 2018	3771	5999	0.63	[0.62; 0.64]
Jacobi, 2016	9	67	0.13	[0.06; 0.24]
Magis, 2017	120	174	0.69	[0.62; 0.76]
Total		18734	0.60	[0.42; 0.77]
Heterogeneity: $\chi^2_7 = 1610.85$ ($P < .001$), $I^2 = 100\%$				
RoB = Low Risk				
Blauvelt, 2020a	1900	4314	0.44	[0.43; 0.46]
Blauvelt, 2020b	872	1797	0.49	[0.46; 0.51]
Bonafede, 2012	230	874	0.26	[0.23; 0.29]
Bonafede, 2013	1408	2775	0.51	[0.49; 0.53]
Cao, 2014	814	1000	0.81	[0.79; 0.84]
Chastek, 2013	341	827	0.41	[0.38; 0.45]
Chastek, 2016	2187	5082	0.43	[0.42; 0.44]
Clemmensen, 2011	147	179	0.82	[0.76; 0.87]
Degli Esposti, 2014	253	378	0.67	[0.62; 0.72]
Doshi, 2016	1489	2707	0.55	[0.53; 0.57]
Esposito, 2013	472	650	0.73	[0.69; 0.76]
Feldman, 2015	1492	4309	0.35	[0.33; 0.36]
Gniadecki, 2011	337	747	0.45	[0.42; 0.49]
Hendrix, 2020	17642	54330	0.32	[0.32; 0.33]
Higa, 2019	1200	2161	0.56	[0.53; 0.58]
Howe, 2014	374	781	0.48	[0.44; 0.51]
Khalid, 2014	108	169	0.64	[0.56; 0.71]
Lee, 2018	1429	2249	0.64	[0.62; 0.66]
Mahlich, 2019	99	176	0.56	[0.49; 0.64]
Malatestinic, 2017	8033	11067	0.73	[0.72; 0.73]
Menter, 2016	2363	3473	0.68	[0.66; 0.70]
Mercadal Orfilia, 2016	91	112	0.81	[0.73; 0.88]
Mocanu, 2019	64	84	0.76	[0.66; 0.85]
Murage, 2019a	1611	2130	0.76	[0.74; 0.77]
Murage, 2019b	646	801	0.81	[0.78; 0.83]
Onsun, 2020	215	247	0.87	[0.82; 0.91]
Pogacsas, 2017	1007	1263	0.80	[0.77; 0.82]
Ross, 2016	33	58	0.57	[0.43; 0.70]
Ruiz-Villaverde, 2020	149	171	0.87	[0.81; 0.92]
Sbidian, 2019	10241	16545	0.62	[0.61; 0.63]
Sruamsiri, 2018	135	205	0.66	[0.59; 0.72]
Svedbom, 2014	287	506	0.57	[0.52; 0.61]
Umezawa, 2013	104	127	0.82	[0.74; 0.88]
van der Reek, 2014	88	116	0.76	[0.67; 0.83]
Verma, 2017	617	882	0.70	[0.67; 0.73]
Wu, 2020	2112	6331	0.33	[0.32; 0.35]
Yiu, 2020	4930	6497	0.76	[0.75; 0.77]
Zagni, 2020	1453	1947	0.75	[0.73; 0.77]
Total		138067	0.63	[0.57; 0.68]
Heterogeneity: $\chi^2_{37} = 17804.35$ ($P < .001$), $I^2 = 100\%$				
Total		156801	0.63	[0.57; 0.68]
Heterogeneity: $\chi^2_{45} = 19429.06$ ($P < .001$), $I^2 = 100\%$				
Test for subgroup differences: $\chi^2_1 = 0.08$ ($P = .78$)				

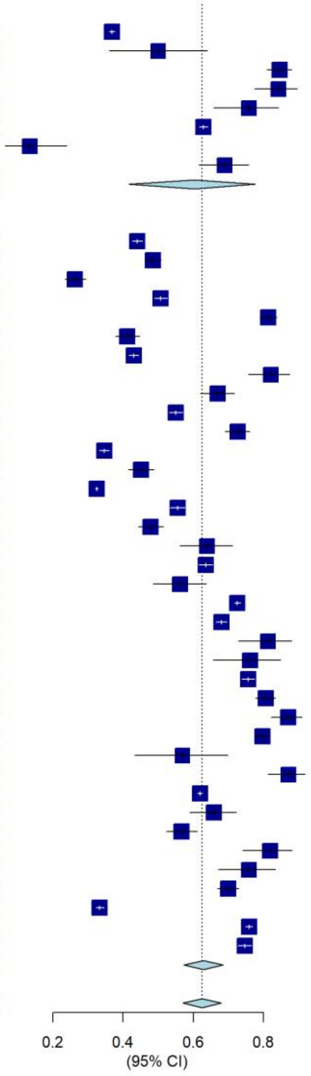

Supplement: Supplementary file 1 [file jcm-11-01506-s001.zip › jcm-1605635-supplementary.pdf]
